# Synthesis, Characterization and Antibacterial Activity of some Novel Thiosemicarbazides, 1,2,4-Triazol-3-thiols and their *S*-substituted Derivatives

**Published:** 2015

**Authors:** Mehdi Kalhor, Mahboobeh Shabani, Iraj Nikokar, Seyedeh Reyhaneh Banisaeed

**Affiliations:** a *Department of Chemistry, Payame Noor University, Tehran, Iran*.; b Laboratory of Microbiology and Immunology of Infectious Diseases, *Guilan Universityof Medical Sciences, Guilan, Iran.*

**Keywords:** Thiosemicarbazide, 1, 2, 4-Triazole-3-thiol, *S*-alkylation, Microorganisms, Antibacterial activity

## Abstract

The thiosemicarbazides 3a-c were appeared by reaction of the corresponding substituted hydrazides 1a-c with allylisothiocyanate 2. Synthesis of some novel 1,2,4-triazole-thiols 4a-c bearing a pyridyl unit using 1-(x-picolinoyl)-4-allyl-thiosemicarbazides (x = 2,3,4) in an alkaline solution, is reported. Also, the *S*-alkylation of triazole derivatives 5-7a-c is described. The structure of the synthesized compounds resulted from the IR, ^1^H and -^13^C NMR spectroscopy and elemental analysis data. The antibacterial studies to all of the synthesized compounds against *B. cereus*, *E. coli*,* P. aeroginosa*,* S. aureus *and* E. faecalis* as MIC values are reported. Some of these compounds such as 7a, 4a and 3a exhibited a good to significant antibacterial activity.

## Introduction

It is well known that thiosemicarbazide derivatives exhibit interesting biological properties such as antitubercular (1), antiviral (2), antimalarial and antibacterial activity (3). These compounds are not only used as a segment in biologically and chemically buildings (4-6), but also as a versatile intermediate for the synthesis of importance heterocycles such as triazoles, thiadiazoles, oxadiazoles and thiazolidinones (7-9).

The fused and pendent 1,2,4-triazoles are a ubiquitous feature of many pharmaceutical products and can be found in marketed drugs such as fluconazole (10), terconazole (11), rizatriptan, alperazolame and triazolame (12). In addition, 1,2,4-triazoles and pyridines (13-15) have attracted the attention of chemists due to attractive biological activities such as antidepressant (16) anti-inflammatory (17), antiviral (18) anticancer (19), anticonvulsant (20,21), antimicrobial (22) and herbicidal (23) properties. On the other hand, incorporating the pyridine ring into active compounds may improve their biological or physiological activities (24). Therefore, the synthesis of novel thiosemicarbazide and triazole derivatives is still interesting for both organic and medicinal chemistry.

In view of these reports and as part of ongoing studies on the synthesis and biological consideration of heterocycles (22,23,25,26), we wish to describe the synthesis of new series of 5-membered heterocyclic titled compounds, bearing the 5-isomeric pyridyl, 4-*N*-allyl and their antibacterial activities.

## Experimental

All chemicals used were purchased from Merck or Fluka. Melting points were determined using an electrothermal digital apparatus and are uncorrected. FT-IR spectra were obtained with a SHIMADZU -IR Presting-21 spectrometer using KBr discs. NMR spectra were recorded on a Bruker (400 or 500 MHz) spectrometer. Chemical shifts (ppm) are referenced to tetramethylsilane (TMS) as internal standard. Elemental analyses were performed with as Elemental Analyzer (Elemental, Vario EL III) at Arak University. Reactions were monitored by thin layer chromatography (TLC). The 2-pyridyl hydrazide 1a was prepared with low improvement following the previously reported procedure (27).

*Biological screening*

The antibacterial activity of synthesized compounds were screened at a concentration of 5 mg/mL against five reference strains of bacteria (Three gram positive as *Bacillus cereus* ATCC 11778, *Staphylococcus aureus* ATCC 25923,* Enterococcus faecalis* ATCC 29212, and two gram negative as *Escherichia coli* ATCC 25922, *pseudomonas aeruginosa* ATCC 27853). Tested compounds were dissolved in dimethyl sulfoxide (DMSO) for the preparation of stock solution. The solvent control was included, although no antibacterial activity has been noted. All samples were tested in triplicate and the average results were recorded. Microbial susceptibility testing of all compounds was carried out by diffusion agar and minimal inhibitory concentration (MIC) methods according to Clinical and Laboratory Standards Institute (CLSI) guideline (28). The suspension of bacteria was adjusted to 0.5 MacFarland Standard (10^6^ c.f.u/mL) and spread over Muller-Hinton Agar. The tested compounds are placed in random well position on the plate, after overnight incubation at 37 °C the zone of inhibition determinate. The antibacterial effects of the compounds that produced ≥ 8mm zone of inhibition were tested quantitatively by Micro broth dilution method for determination of MIC value that were defined as lowest concentration of compound for inhibition growth of tested bacteria. In this method concentration of 512, 256, 128, 64, 32, 16, 8 μg/mL were used for all bacteria in per disc and incubated the same conditions.

*General procedure for the synthesis of thiosemicarbazides (3a-c)*

A solution of allyl isothiocyanate, 2, (0.01 mol, 0.99 g) in ethanol (10 mL) was added to a solution of pyridyl acid hydrazide 1(a-c), (0.01 mol, 1.37 g) in absolute ethanol (15 mL) with stirring. The reaction mixture was refluxed for 4-5 h. The solution was cooled to ambient temperature, and the precipitate was filtered to give the crude product, which was then recrystallized from appropriate solvent to give pure compounds 3a–c.

*4-Allyl-1-picolinoyl thiosemicarbazide (3a)*

Reaction time: 4h, m.p. 182-183 ºC, 86% yield, recrystallization solvent: EtOH; FT-IR (KBr, ν_max_): 3195 (N-H), 3088, 2964 (C-H), 1666 (C=O), 1587, 1542, 1512, 1457, 1284 (C=N, C=C), 1222 (C=S), 1159, 1002, 920, 746, 685 cm^-1^;^ 1^H-NMR (acetone-*d*_6_, 400MHz): δ = 4.24- 4.28 (m, 2H, N-CH_2_), 5.02 (d, 1H, *J *= 10.1 Hz, H_cis_-CH=CH_-_), 5.16 (d, 1H, *J *= 16.8 Hz, H_trans_-CH=CH_-_), 5.84-5.94 (m, 1H, C=CH-C), 7.63-7.66 (m, 1H, H_pyr_), 7.95 (br, 1H, NH), 8.01-8.06 (m, 1H, H_pyr_), 8.1 (d, 1H, *J *= 7.6 Hz, H_pyr_), 8.65-8.67 (m, 1H, H_pyr_), 8.70 (br, 1H, NH), 10.20 (s, 1H, NH) ;^13^C-NMR (aceton-*d*_6__,_ 100MHz): δ = 46.4 (C aliphatic), 115.0, 122.3, 126.9, 134.5, 137.6, 148.5, 152.2 (C-Allyl and Aryl), 166.1 (C=S), 184.2 (C=O) ppm; Anal. Calcd. for C_10_H_12_N_4_OS: C, 50.83; H, 5.12; N, 23.71; S, 13.57; found: C, 51.01; H, 5.13; N, 23.76; S, 13.63%.

*4-Allyl-1-nicotinoyl thiosemicarbazide (3b)*

Reaction time: 5h, m.p. 182-183 ºC, 62% yield, recrystallization solvent: EtOH; FT-IR (KBr, ν_max_): 3226, 3170 (N-H), 2980 (C-H), 1693 (C=O), 1676, 1540, 1514, 1273 (C=N, C=C), 1234 (C=S), 1022, 894, 700, 567 cm^-1^;^ 1^H-NMR (acetone-*d*_6_, 400MHz): δ = 4.24-4.28 (m, 2H, N-CH_2_), 5.02 (d, 1H, *J *= 10.4 Hz, H_cis_-CH=CH), 5.14 (d, 1H, *J* = 17.2 Hz, H_trans_-CH=CH_-_), 5.83- 5.93 (m, 1H, C=CH-C), 7.51-7.55 (m, 1H, H_pyr_), 8.09 (br, 1H, NH), 8.28 (d, 1H, *J *= 8.0 Hz, H_pyr_), 8.64 (br, 1H, NH), 8.76 (d, 1H, *J* = 5.6 Hz, H_pyr_), 9.13 (s, 1H, H_pyr_), 9.93 (br, 1H, NH) ppm; ^13^C-NMR (acetone-*d*_6__, _100MHz): δ = 46.4 (C-aliphatic), 115.0, 123.4, 128.2, 134.5, 135.2, 148.8, 152.6 (C-Allyl and Aryl), 165.1 (C=S), 183.7 (C=O) ppm; Anal. Calcd. for C_10_H_12_N_4_OS: C, 50.83; H, 5.12; N, 23.71; S, 13.57; found: C, 50.47; H, 5.14; N, 23.65; S, 13.52%.

*General procedure for the synthesis of 1,2,4-triazole-3-thiols 4(a-c)*

A solution of thiosemicarbazide, 3(a-c), (5 mmol, 1.180 g) in 2N NaOH (10 mL) was refluxed for 2-3 h. The resulting solution was cooled to room temperature and acidified (pH = 3) with 2N HCl. The precipitate was filtered and washed with water and ethanol. The obtained compound was dried and crystallized from suitable solvent to give compound 4(a-c).

4-Allyl-5-(pyridin-2-yl)-4*H*-[1,2,4]triazole-3-thiol (4a). Reaction time: 2 h, m.p. 182-183 ºC, 84% yield, recrystallization solvent: DMF:EtOH (1:2); FT-IR (KBr, ν_max_): 2927 (C-H), 2773 (SH), 1585, 1546, 1500, 1463, 1396, 1336 (C=N, C=C), 1290, 1268, 995, 771, 698, 569 cm^-1^;^ 1^H-NMR (CDCl_3_, 400MHz): δ = 5.14-5.20 (m, 2H, N-CH_2_), 5.39 (d, 2H, *J* = 5.6 Hz, CH_2_=C-C), 5.92-6.02 (m 1H,C=CH-C), 7.39-7.43 (m, 1H, H_pyr_), 7.82-7.86 (t, *J* = 7.6 Hz, 1H, H_pyr_), 8.04 (d, 1H, *J* = 7.2 Hz, H_pyr_), 8.68-8.69 (m, 1H, H_pyr_), 12.70 (s, 1H, SH or NH) ppm; the SH or NH proton disappeared on D_2_O addition; ^13^C-NMR (CDCl_3_, 100 MHz): δ = 47.5 (C aliphatic), 118.5, 123.3, 124.9, 131.4, 137.2, 146.1, 149.0, 149.2 (C-Allyl and Aryl), 168.5 (C=S) ppm; Anal. Calcd. for C_10_H_10_N_4_S: C, 55.02; H, 4.62; N, 25.67; S, 14.69; found: C, 55.31; H, 4.60; N, 25.57; S, 14.66%.

*4-Allyl-5-(pyridin-3-yl)-4H-[1,2,4]triazole-3-thiol (4b)*

Reaction time: 3 h, m.p. 177-180 ºC, 78% yield, recrystallization solvent: DMF: EtOH (1:2); FT-IR (KBr, ν_max_): 2715 (SH), 1558, 1483, 1436, 1420, 1350, 1307 (C=N, C=C), 1262, 1188, 945, 810, 704, 615 cm^-1^; ^1^H-NMR (acetone-*d*_6_, 400 MHz): δ = 4.81- 4.83 (m, 2H, N-CH_2_), 5.00 (d, 1H, *J* = 17.6 Hz, H_trans_-C=C-), 5.18 (d, 1H, *J* =10.4 Hz, H_cis_-C=C-) 5.90-6.00 (m, 1H,C=CH-C), 7.58-7.62 (m, 1H, H_pyr_), 8.15 (d, *J* = 8.4, 1H, H_pyr_), 8.78 (d, *J* = 3.2, 1H, H_pyr_), 8.93 (s, 1H, H_pyr_), 12.95 (s, 1H, SH or NH) ppm; ^13^C-NMR (aceton-*d*_6_, 100 MH): δ = 46.3 (C aliphatic), 117.1, 122.9, 123.6, 131.8, 135.8, 149.1, 151.6, 153.2 (C-Allyl and Aryl), 168.5 (C=S) ppm; Anal. Calcd. for C_10_H_10_N_4_S: C, 55.02; H, 4.62; N, 25.67; S, 14.69; found: C, 55.31; H, 4.65; N, 25.77; S, 14.71%.

*General procedure for the synthesis of S-substituted-1,2,4-triazoles 5-7(a-c)*

To a solution of triazole 4 (a-c) (3 mmol, 0.65 g) in absolute ethanol (20 mL), ethyl chloroacetate, iodoacetamide or chloroacetic acid (6 mmol) was added. The mixture was refluxed (for 6,7a-c in room temperature) under stirring for 1-5 h in the presence of KOH (6 mmol, 0.336 g). Then, the solvent was removed under reduced pressure to give the solid product. The crude product was recrystallized from suitable solvent to give compound 5-7 (a-c).

*Ethyl 2-((4-allyl-5-(pyridin-2-yl)-4H-1,2,4-triazol-3-yl)thio)acetate (5a)*

Reaction time: 2 h, m.p. 56-58 ºC, 66% yield, recrystallization solvent: H_2_O:EtOH (1:1); FT-IR (KBr, ν_max_): 3047, 2981 (CH), 1725 (C=O), 1589, 1489, 1446, 1425, 1411, 1311(C=N, C=C), 1201, 1176 (C-O), 796, 704 (S-C) cm^-1^; ^1^H-NMR (CDCl_3_, 400 MHz): δ = 1.26 (t, *J* = 7.2 Hz, 3H, CH_3_), 4.19 (q, 2H , *J* = 6.8 Hz, O-CH_2_), 4.13 (s, 2H, S-CH_2_), 5.07 (d, *J* = 17.2 Hz, 1H, H_trans_-CH=CH-), 5.18 (d, *J* = 10.4 Hz, 1H, H_cis_-CH=CH-), 5.28 (m, 2H, N-CH_2_), 5.94-6.01 (m,1H, C=CH-C), 7.34 (m,1H, H_pyr_), 7.79 (t, *J *= 8.0 Hz, 1H, H_pyr_), 8.27 (d, *J* = 8.0 Hz, 1H, H_pyr_), 8.61-8.63 (m, 1H, H_pyr_) ppm; ^13^C-NMR (CDCl_3_, 100 MHz): δ= 14.1, 35.3, 47.9, 62.0 (C aliphatic), 118.1, 123.2, 124.0, 132.1, 136.9, 147.7, 148.7, 151.9, 153.0 (C-Allyl and Aryl), 168.3 (C=O) ppm; Anal. Calcd. for C_14_H_16_N_4_O_2_S: C, 55.25; H, 5.30; N, 18.41; S, 10.53; found: C, 55.55; H, 5.29; N, 18.43; S, 10.53%.

*Ethyl 2-((4-allyl-5-(pyridin-3-yl)-4H-1,2,4-triazol-3-yl)thio)acetate (5b)*

Reaction time: 1.5 h, m.p. 82-84 ºC, 57% yield, recrystallization solvent: H_2_O:EtOH (1:1); FT-IR (KBr, ν_max_): 3046, 2937 (C-H), 1739 (C=O), 1600, 1462, 1572, 1423, 1373, 1301 (C=N, C=C), 1186 (C-O), 1163, 711 (C-S) cm^-1^; ^1^H-NMR (DMSO-*d*_6_, 400 MHz): δ= 1.16 (t, 3H, *J* = 6.8 Hz, 3H, CH_3_), 4.09 (m, 4H, O-CH_2 _and S-CH_2_), 4.68 (d, *J* = 4.4 Hz, 2H, N-CH_2_), 4.81 (d, 1H, *J *= 16.4, H_trans_-CH=CH-), 5.22 (d, *J* = 9.6 Hz, 1H, H_cis_-CH=CH-), 5.92-6.02 (m, 1H, C=CH-C), 7.57 (m,1H, H_pyr_), 8.03 (m, 1H, H_pyr_), 8.73 (d, *J* = 6.4 Hz, 1H, H_pyr_), 8.82 (s, 1H, H_pyr_) ppm; ^13^C-NMR (DMSO-*d*_6_, 100 MHz): δ = 14.4, 35.0, 47.0, 61.7 (C aliphatic), 117.7, 123.6, 124.4, 132.7, 136.1, 148.9, 151.3, 151.4, 153.4 (C-Allyl and Aryl), 168.6 (C=O) ppm; Anal. Calcd. for C_14_H_16_N_4_O_2_S: C, 55.25; H, 5.30; N, 18.41; S, 10.53; found: C, 55.62; H, 5.31; N, 18.39; S, 10.49%.

*2-((4-Allyl-5-(pyridin-2-yl)-4H-1,2,4-triazol-3-yl)thio)acetamide (6a)*

Reaction time: 5 h, m.p. 182-184 ºC, 65% yield**,** recrystallization solvent: H_2_O:EtOH (1:1); FT-IR (KBr, ν_max_): 3300, 3142 (NH_2_), 3072, 2976 (C-H), 1705 (C=O), 1639, 1587, 1470, 1446, 1417 (C=N, C=C), 923, 781 (S-C), 698 (C-S) cm^-1^ ;^1^H-NMR (DMSO-*d*_6_, 500MHz): δ = 3.90 (s, 2H, S-CH_2_), 4.83 (d, 1H, *J* = 17.29 Hz, H_trans_-CH=CH- ), 5.08 (d, 1H, *J *= 10.5 Hz, H_cis_-CH=CH), 5.14 (d, *J *= 5.5 Hz, 2H, N-CH_2_), 5.89-5.96 (m, 1H, C=CH-C), 7.20 (br, 1H, NH), 7.45-7.47 (m, 1H, H_pyr_), 7.63 (br, 1H, NH), 7.92 (m, 1H, H_pyr_), 8.07 (d, 1H, *J* = 7.9 Hz, H_pyr_), 8.64 (d, *J* = 4.8 Hz, 1H, H_pyr_) ppm; ^13^C-NMR (DMSO-*d*_6_, 125MHz): δ = 36.1, 47.6 (C aliphatic), 117.04, 122.7, 124.2, 128.7, 132.8, 137.2, 148.9, 152.7, 153.4 (C-Allyl and Aryl), 168.5 (C=O) ppm; Anal. Calcd. for C_12_H_13_N_5_OS: C, 52.35; H, 4.76; N, 25.44; S, 11.65; found: C, 52.21; H, 4.76; N, 25.28; S, 11.62**%**.

*2-((4-Allyl-5-(pyridin-2-yl)-4H-1,2,4-triazol-3-yl)thio)acetamide (6b)*

Reaction time: 5h, m.p. 161-163 ºC, 50% yield**,** recrystallization solvent: H_2_O:EtOH (1:1); FT-IR (KBr, ν_max_): 3346, 3188 (NH_2_), 1690 (C=O), 1450, 1394, 1222 (C=N, C=C), 979, 815 (S-C), 621 cm^-1^; ^1^H-NMR (DMSO-*d*_6_, 500MHz): δ = 3.91 (s, 2H, S-CH_2_), 4.64 (d, 2H, *J* = 4.3 Hz, N-CH_2_), 4.78 (d, 1H, *J* = 17.3 Hz, H_trans_-CH=CH-), 5.17 (d, *J* = 10.5 Hz, 1H, H_cis_-CH=CH-), 5.89-5.96 (m, 1H, C=CH-C), 7.20 (br, 1H, NH), 7.52-7.55 (m, 1H, H_pyr_), 7.6 (br, 1H, NH), 7.99 (d, 1H, *J* = 7.9 Hz, H_pyr_), 8.68 (d, 1H, *J* = 4.3 Hz, H_pyr_), 8.78 (s, 1H, H_pyr_) ppm; ^13^C-NMR (DMSO-*d*_6_, 125MHz): δ = 37.1, 47.0 (C aliphatic), 117.7, 123.7, 124.3, 132.8, 136.1, 148.9, 151.4, 152.0, 153.2 (C-Allyl and Aryl), 169.0 (C=O) ppm; Anal. Calcd. for C_12_H_13_N_5_OS: C, 52.35; H, 4.76; N, 25.44; S, 11.65; found: C, 52.15; H, 4.74; N, 25.49; S, 11.67**%**.

*2-((4-Allyl-5-(pyridin-2-yl)-4H-1,2,4-triazol-3-yl)thio)acetamide (6c)*

Reaction time: 5 h, m.p. 174-176 ºC, 83% yield, recrystallization solvent: H_2_O:EtOH (1:1); FT-IR (KBr, ν_max_): 3338, 3186 (NH_2_), 1690 (C=O), 1604, 1454, 1427, 1404, 1226 (C=N, C=C), 981, 835, 769 (S-C), 698 cm^-1 ^; ^1^H-NMR (DMSO-*d*_6_, 500MHz): δ = 3.93 (s, 2H, S-CH_2_), 4.70 (d, *J* = 4.4 Hz, 2H, N-CH_2_), 4.80 (d, 1H, *J* = 17.2 Hz, H_trans_-CH=CH-), 5.19 (d, 1H, *J* = 10.5 Hz, H_cis_-CH=CH-), 5.92-5.99 (m, 1H, C=CH-C), 7.20 (br, 1H, NH), 7.61 (m, 2H, H_pyr_), 7.66 (br, 1H, NH), 8.70 (q, *J* = 1.6 Hz, *J* = 2.9 Hz, 2H, H_pyr_) ppm ;^ 13^C-NMR (DMSO-*d*_6_, 125MHz): δ = 37.1, 47.1 (C aliphatic), 117.7, 122.4, 124.3, 132.7, 134.6, 148.9, 150.9, 152.7, 153.4 (9 C Allyl and Aryl), 169.0 (C=O) ppm; Anal. Calcd. for C_12_H_13_N_5_OS: C, 52.35; H, 4.76; N, 25.44; S, 11.65; found: C, 52.20; H, 4.76; N, 25.40; S, 11.69**%**. 

*2-((4-Allyl-5-(pyridin-2-yl)-4H-1,2,4-triazol-3-yl)thio)acetic acid (7a)*

Reaction time: 4 h, m.p. 109-111 ºC, 75% yield, recrystallization solvent: DMF:EtOH (8:2); FT-IR (KBr, ν_max_): 3380 (OH), 3077, 2984 (C-H), 1682 (C=O), 1615, 1586, 1471, 1422, 1388 (C=N, C=C), 1215, 999, 924, 790 (S-C) cm^-1^; ^1^H-NMR (DMSO-*d*_6_, 500MHz): δ = 3.80 (s, 2H, S-CH_2_), 4.78 (d, *J* = 16.5 Hz, 1H, H_trans_-CH=CH-), 5.16 (br s, 3H, H_cis_-CH=CH- and N-CH_2_), 5.95 (m, 1H, C=CH-C), 7.48 (br s,1H, H_pyr_), 7.96-8.10 (br d, 2H, H_pyr_), 8.66 (br s, 1H, H_pyr_), ppm; ^13^C-NMR (DMSO-*d*_6_, 125MHz): δ = 40.7, 47.4 (C aliphatic), 117.4, 123.1, 124.7, 133.3, 138.0, 147.7, 149.4, 152.2, 154.6, 169.7 (C=O) ppm; Anal. Calcd. for C_12_H_12_N_4_O_2_S: C, 52.16; H, 4.38; N, 20.28; S, 11.60; found: C, 52.35; H, 4.39; N, 20.30; S, 11.62%.

*2-((4-Allyl-5-(pyridin-3-yl)-4H-1,2,4-triazol-3-yl)thio)acetic acid (7b)*

Reaction time: 5 h, m.p. 90 ºC, 60% yield, recrystallization solvent: DMF:EtOH (8:2); FT-IR (KBr, ν_max_): 3384 (OH), 1682 (C=O), 1586, 1482, 1453, 1439 (C=N, C=C), 1229 (C-O), 1022, 943, 778 (S-C) cm^-1^; ^1^H-NMR (DMSO-*d*_6_, 500MHz): δ = 3.75 (s, 2H, S-CH_2_), 4.66 (s, 2H, N-CH_2_), 4.78 (d, *J* = 17.5 Hz, 1H, H_trans_-CH=CH-), 5.20 (d, *J* = 10.5 Hz, 1H, H_cis_-CH=CH-), 5.93-5.99 (m, 1H, C=CH-C), 7.56 (q, *J* = 5.0 Hz, 1H, H_pyr_), 8.03 (t, *J *= 7.8 Hz, 1H, H_pyr_), 8.71 (q, *J* = 4.8 Hz, 1H, H_pyr_), 8.82 (s, 1H, H_pyr_) ppm; ^13^C-NMR (DMSO-*d*_6_, 125MHz): δ = 41.1, 46.8 (C aliphatic), 117.3, 124.0, 124.3, 133.0, 135.9, 148.8, 151.1, 152.5, 154.0, 169.0 (C=O) ppm; Anal. Calcd. for C_12_H_12_N_4_O_2_S: C, 52.16; H, 4.38; N, 20.28; S, 11.60; found: C, 52.41; H, 4.36; N, 20.24; S, 11.64%.

*2-((4-Allyl-5-(pyridin-4-yl)-4H-1,2,4-triazol-3-yl)thio)acetic acid (7c)*

Reaction time: 4 h, m.p. 240-242 ºC, 85% yield, recrystallization solvent: DMF:EtOH (8:2); FT-IR (KBr, ν_max_): 3382 (OH), 1684 (C=O), 1614, 1457, 1387, 1331 (C=N, C=C), 1263, 1227 (C-O), 985, 831 (S-C) cm^-1^; ^1^H-NMR (DMSO-*d*_6_, 500MHz): δ = 3.79 (s, 2H, S-CH_2_), 4.72 (s, 2H, N-CH_2_), 4.78 (d, *J* = 17.5 Hz, 1H, H_trans_-CH=CH-), 5.22 (d, *J* = 10.5 Hz, 1H, H_cis_-CH=CH-), 5.96-6.03 (m, 1H, C=CH-C), 7.64 (d, *J* = 6.0 Hz, 2H, H_pyr_), 8.72 (d, *J *= 6.0 Hz, 2H, H_pyr_) ppm; ^13^C-NMR (DMSO-*d*_6_, 125MHz): δ = 40.8, 46.9 (C aliphatic), 117.4, 122.3, 132.8, 134.9, 150.8, 152.8, 154.6, 169.6 (C=O) ppm; Anal. Calcd. for C_12_H_12_N_4_O_2_S: C, 52.16; H, 4.38; N, 20.28; S, 11.60; found: C, 51,97; H, 4.39; N, 20.30; S, 11.60%.

## Results and Discussion

*Synthesis*

**Figure 1:**
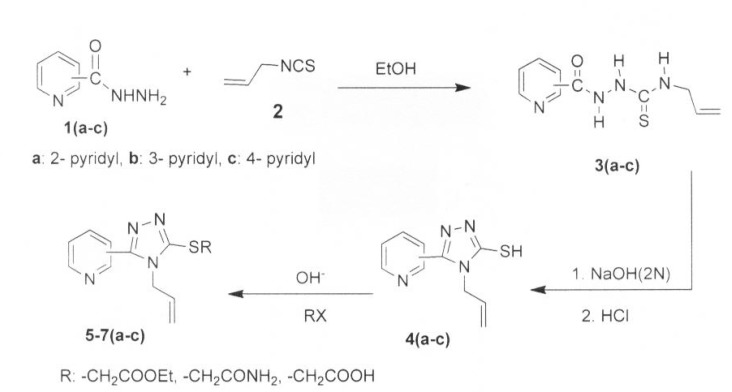
Synthetic route of compound 3-7 (a-c).

The synthesis of the titled compounds is illustrated in Figure 1. The preparation of thiosemicarbazides 3a-c was achieved by reaction of the isomeric pyridine carboxylic acid hydrazides 1a-c with allylisothiocyanate 2. Thiosemicarbazide derivatives 3a-c underwent an intramolecular cyclization under basic conditions to produce 1,2,4-triazoles 4a-c in high yields. The reaction yields for 4c (94%) is higher than other respective isomers, which may attributed to the stereo-electronic effects of the nitrogen atom of the pyridine ring and also due to the more symmetrical structure of these products. The *S*-alkylated 1,2,4-triazoles, esters 5a–c, acetamides 6a-c and acetic acids 7a-c were also prepared by reaction of 1,2,4-triazole-3-thioles 4a-c and corresponding reagents, as previously reported (6,21).

The IR spectra of thiosemicarbazides 3a-c showed characteristic absorption bonds at 1222-1234, 1666-1693, and 3170-3226 cm^-1^ for the -C=S, C=O and –NH stretching vibration respectively. The *trans *and *cis* protons in allyl group for compounds 3a,b appeared as doublet signals at the region between 5.02-5.16 ppm with *J* = 16.8-17.5 and 10.1-10.4 Hz. The ^1^H NMR spectra of 3a,b showed singlet signals at 7.95-10.20 ppm due to the resonance of -NH-CS-NH and -CO-NH protons, which disappeared upon D_2_O addition. The ^13^C NMR spectra of 3a,b showed ten signals including signals at 165.1-166.1 ppm for the -C=S and signals at 187.7-184.2 ppm for the –C=O group.

The ^1^H NMR spectra of triazole-3-thiols 4a-c showed singlets at 12.70-12.95 ppm attributed to the resonance of the SH or NH protons, which disappeared upon D_2_O addition. In the ^13^C NMR spectra of 4a-c, the appearance of signals at the region 168.5 ppm attributed to the carbon resonance of the C=N or C=S group in triazole rings which is in support of the expected structures. 

 In the ^1^H NMR spectra of 5-7a-c, the absence of the -SH resonance and the appearance of a singlet in the aliphatic region, related to the resonance of the –SCH_2_- group, supports the formation of the alkylated products. All other required peaks in target new compounds appeared in exhibited region of the spectrums. However, the acidic proton (COOH) of compounds 7a-c was not observed in the spectrum, probably due to the effect of exchange of this acidic proton with deuterium of small amounts of D_2_O, which is present in DMSO-*d*_6_, as a solvent (29).

*Antibacterial activities*

 Applying the agar plate diffusion technique (30), all of newly synthesized compounds were screened *in-vitro* for antimicrobial activities against five pathogenic bacteria. The results of the bioassay are given in Table 1. A cursory view of the data indicates that some of the compounds 4,6,7a and specially 3a exhibit a moderate to good activity against four bacteria with gram positive (*Bacillus cereus*,* Staphylococcus aureus*) and negative (*Escherichia coli* , *pseudomonas aeruginosa* ) strain. It is considerable that thiosemicarbazide 3a was found to be more active against three microorganisms than Gentamicin, which is a known antimicrobial drug. Therefore, this compound can have the potential to be good antibacterial candidate that the research is ongoing in this regard. Although compounds 7a and 7c has shown the good activity against *E. faecalis*, the other compounds do not show any antibacterial activity. 

**Table 1:** Antibacterial activities of chemical compounds 1-7(a-c) *(zone of inhibition in mm).*

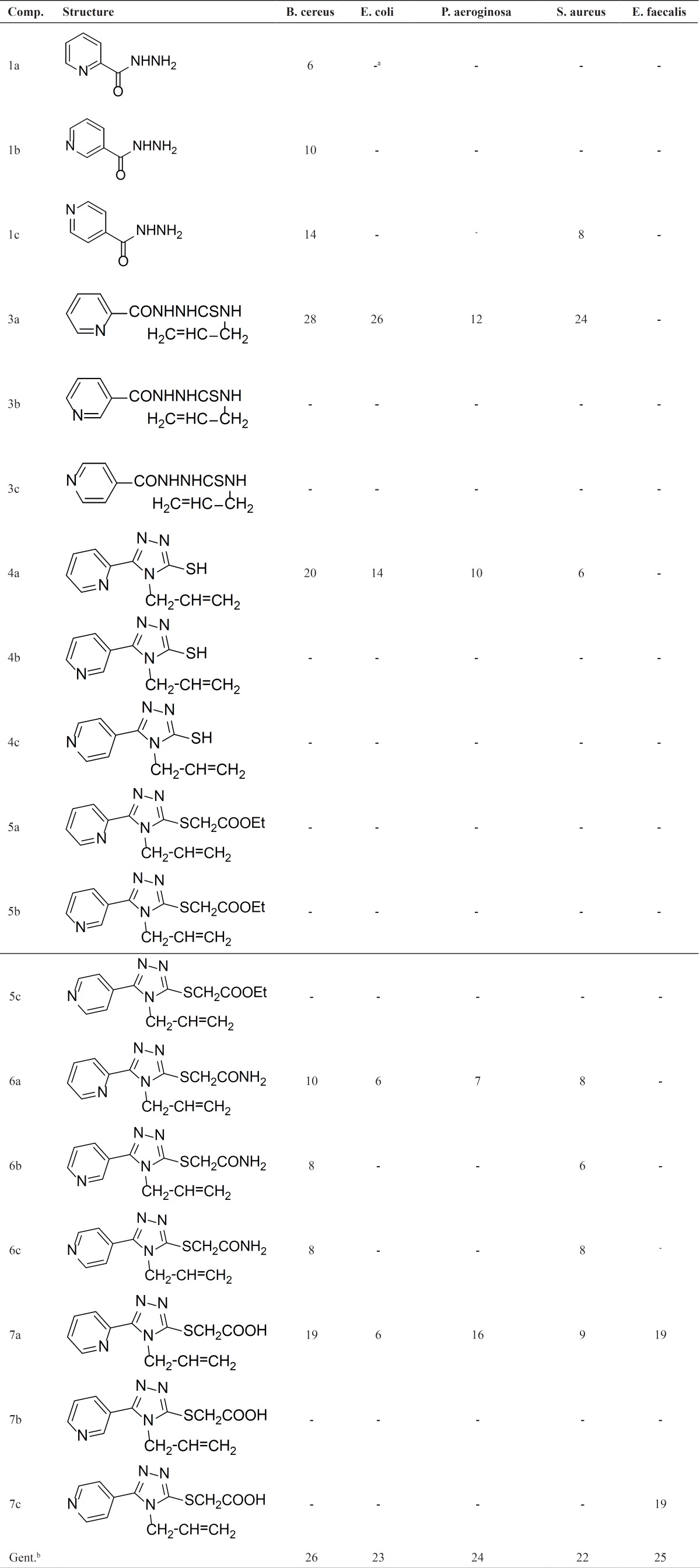

Dimethyl sulfoxide (DMSO) only, control for compounds and references.

^a^ Not active

^b^ *Gentamicin*: reference compound.

In addition, investigation of the minimum inhibitory concentration (MIC) values of the potent derivatives against five microorganisms was performed and the results are presented in Table 2. Just as was predicated, compound 3a indicates the highest bactericidal activity (16-64 µg/mL) against all organism tests (except for *E. faecalis*). 

**Table 2:** Minimum inhibitory concentration (MIC) of the selected compounds against microbial strains (µg/mL)

**Compound**	***B. cereus***	***E. coli***	***P. aeroginosa ***	***S. aureus***	***E. faecalis***
1b	>512	NT	NT	NT	NT
1c	>512	NT	NT	>512	NT
3a	64	32	64	16	NT
4a	64	128	>512	NT	NT
6a	256	NT	NT	128	NT
6b	512	NT	NT	NT	NT
6c	512	NT	NT	>512	NT
7a	128	NT	128	>512	128
7c	NT	NT	NT	NT	128
Gentamicin^a^	2	1	2	1	8

Disc diffusion method used to determine the MICs (30). DMSO only, control for compounds

* NT *not tested

a Reference compound
